# Catastrophic health expenditures of households living with pediatric leukemia in China

**DOI:** 10.1002/cam4.3317

**Published:** 2020-07-22

**Authors:** Mingjie Sui, Xueyun Zeng, Wan Jie Tan, Sihai Tao, Rui Liu, Bo Liu, Wenrui Ma, Weidong Huang, Hongjuan Yu

**Affiliations:** ^1^ School of Health Management Harbin Medical University Harbin China; ^2^ Duke‐NUS Medical School National University of Singapore Singapore Singapore; ^3^ North China University of Science and Technology Hebei China; ^4^ School of Public Health The University of Queensland Brisbane Qld Australia; ^5^ Department of Hematology The First Affiliated Hospital of Harbin Medical University Harbin China

**Keywords:** cancer, catastrophic health expenditure, economic burden, leukemia, pediatric patients

## Abstract

**Background:**

Leukemia can create a significant economic burden on the patients and their families. The objective of this study is to assess the medical expenditure and compensation of pediatric leukemia, and to explore the incidence and determinants of catastrophic health expenditure (CHE) among households with pediatric leukemia patients in China.

**Methods:**

A cross‐sectional interview was conducted among households living with pediatric leukemia using a questionnaire in two tertiary hospitals. CHE was defined as out‐of‐pocket (OOP) payments that were greater than or equal to 40% of a household's capacity to pay (CTP). Chi‐square tests and logistic regression analysis were performed to identify the determinants of CHE.

**Results:**

Among 242 households living with pediatric leukemia, the mean OOP payment for pediatric leukemia healthcare was $9860, which accounted for approximately 35.7% of the mean household's CTP. The overall incidence of CHE was 43.4% and showed a downward trend with the lowest income group at 69.0% to the highest income group at 16.1%. The logistic regression model found that medical insurance, frequency of hospital admissions, charity assistance, and income level were significant predictors of CHE.

**Conclusion:**

The results revealed that pediatric leukemia had a significant catastrophic effect on families, especially those with lower economic status. The occurrence of CHE in households living with pediatric leukemia could be reduced by addressing income disparity. In addition, extending coverage and improving compensation from medical insurance could also alleviate CHE. Some other measures that can be implemented are to address the barriers of charity assistance for vulnerable groups.

## Graphical abstract

Leukemia had a significant catastrophic effect on families, especially those with lower economic status. The occurrence of catastrophic health expenditure (CHE) in households living with pediatric leukemia could be reduced by addressing income disparity. In addition, extending coverage and improving reimbursement from medical insurance could also alleviate CHE. Some other measures that can be implemented are to address the barriers of charity assistance for vulnerable groups.

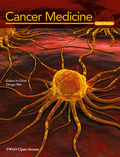

## 1 INTRODUCTION

Leukemia, which is the malignancy of blood cells, is the most prevalent form of cancer in children and adolescents, and is the leading cause of childhood mortality around the world.^1^, ^2^, ^3^ It is recently estimated that 150 500 children were diagnosed with leukemia and 48 800 of the patients died from this disease worldwide in 2017.^4^ Leukemia constitutes the largest percentage of categorized childhood cancer Disability Adjusted Life Year (DALY) globally, with 34.1% of all childhood cancer DALYs (11.5 million) attributable to leukemia.^4^

In China, 35.6 out of every million children under 18 years of age were diagnosed with leukemia, which translates to about 8943 new cases of leukemia in 2015.^1^ It is also the leading cause of childhood deaths in China, with a mortality rate of 15 deaths per million children.^1^ However, with improvements in therapy and early diagnosis over the past few decades, patients with leukemia now have longer life expectancies and improved quality of life.^2^, ^4^, ^5^, ^6^ These advances in treatment have also increased the 5‐year relative survival rates of patients, reaching to around 85% in developed countries and 70.5% to 80% in China.^1^, ^7^, ^8^

Due to the prolonged length of stay in hospitals and the need for specialized facilities and care, children and adolescents with pediatric cancer require a significantly greater amount of healthcare resources than patients with other pediatric health conditions.^9^, ^10^ Aside from medical bills such as diagnostic tests, chemotherapy, and hospitalization, the financial costs for treating pediatric cancer also include hidden non‐medical expenses, such as transport, accommodation, and loss of parental income. Although the compensation such as funded by the medical insurance or government can release the economic burden of households with pediatric cancer to the extent,^11^, ^12^ the rest of medical cost mainly assumed by the family through Out‐Of‐Pocket (OOP) payments, which is defined as the expenditure incurred by an individual and/or their families to secure or maintain access to health services.^13^ When a household must reduce its basic expenses over a period of time in order to cope with OOP payments, it is viewed as a catastrophic health expenditure (CHE). CHE is an index associated with the proportion of OOP payments that exceed a given threshold of the total household income (eg, 10%)^14^, ^15^ or the total household expenditures (eg, 40%) within a given year.^16^

CHE may expose households to a significant risk of impoverishment and can result in economic burden for both developing and developed countries.^17^ Due to the profound effects of CHE on households living with cancer, CHE has received growing research attention over the recent years. In Korea, the incidence of CHE in households living with cancer reached 37.2% with a threshold level of 10%.^18^ In the ASEAN Costs In Oncology (ACTION) study which covered eight counties in the Association of Southeast Asian Nations (ASEAN), findings revealed that over 75% of the patients with cancer experienced financial catastrophe within a year.^19^ In China, two recent studies reported that the incidence of CHE resulting from cancer was 42.78%^20^ and up to 95%,^21^ with 40% as the threshold level. While these studies reveal the financial burden of cancer treatment on a household, existing literature on CHE has only been conducted in the adult cancer population. In a minority of the studies that focused on pediatric cancer, CHE has not been investigated. For example, one study utilized the Family Impact Scale (FIS) to evaluate the impact and financial costs of childhood cancer in Australian families.^22^ In another study, it was reported that the economic impact of advanced pediatric cancer on families in the United States was significant across all income levels, where those from poorer families incurred more severe losses.^9^ Yet, the study did not evaluate CHE resulting from pediatric cancer. In light of the paucity of information regarding CHE among households living with pediatric cancer, this study, therefore, aims to: (a) describe medical expenditure and compensation of pediatric leukemia, (b) assess the incidence of CHE in households living with pediatric leukemia, and (c) explore the determinant factors associated with CHE.

## 2 METHODS

### 2.1 Study design

A cross‐sectional survey was conducted in Heilongjiang, a northeast province in China with a population size of 37.51 million in 2018.^23^ Child‐caregiver dyads consisting of pediatric patients and their family caregivers (FCs) were recruited from two tertiary hospitals located in Harbin, the capital of Heilongjiang province, based on the purposeful sampling. These two hospitals were selected as they are serving as the major referral centers for pediatric leukemia patients across the entire province. Patients must have a diagnosis of leukemia and aged between 0 and 18 in order to participate in the study, while FCs need to be the immediate family member of the patient (eg, mother) and is familiar with the family environment. Prior to participant recruitment, ethics approval was obtained from the Ethics Committee of Harbin Medical University (No. 18HMUDQZH052) from January 2018 to February 2019.

### 2.2 Measures

A draft questionnaire was constructed after integrating previous analogous studies^21^, ^24^ with the opinions of five experts: two health economists, one psychologist, one leukemia doctor, and one sociologist. A pilot survey involving 10 FCs was then conducted and based on the reviews provided by the FCs, further amendments to the items and structure of the draft questionnaire were made. The modified version of the standardized questionnaire comprised of items on demographic profile, household information, financial cost of cancer treatment, compensation on treatment, and clinical information (items in the questionnaire are presented in Appendix A1). The questionnaire was found to have a Cronbach's alpha coefficient of 0.87 in the present study. Information collected on the dyads demographic profile included gender, age, education, and place of residence. Household information included family size, debt, and total annual household income and expenditure. Debt is defined as the amount of money borrowed from individuals outside of the household, such as relatives and friends, to cover the medical expenses incurred over the year. As for information regarding the medical cost of leukemia treatment, medical bills such as outpatient and inpatient care, self‐procurement of drugs, as well as non‐medical expenditure including transportation, accommodation, and nutritional food, were collected. With regard to the compensation provided for medical expenditure, information on compensation from medical insurance was collected as suggested in prior studies.^25^, ^26^, ^27^, ^28^ Medical Insurance for Urban Residents (MIUR) in China covered the treatment of pediatric leukemia for patients who reside in urban areas, while the New Cooperative Medical Scheme (NCMS) covered those who reside in the rural areas. Besides these governmental medical insurances (MIUR/NCMS), information was also collected regarding the compensation from the Commercial Medical Insurance (CMI), a sort of private insurance. Other sources of compensation, such as government subsidy, support from relatives and friends, and charity assistance were also examined. Charity assistance is a relatively new funding method organized by charity organizations or through crowdfunding on social media, which aims to provide financial support to patients of leukemia.

In addition, clinical information such as the type of leukemia diagnosed (AML: acute myeloid leukemia; ALL: acute lymphocytic leukemia; CLL: chronic lymphocytic leukemia; CML: chronic myeloid leukemia), treatment method, and frequency of annual hospital admissions were also obtained.

### 2.3 Procedure

Child‐caregiver dyads were approached by members of the research team and were invited to participate in the study. Upon consent to participate in the study, an informed consent form was completed by the FC of the patient. The FC was then led to a private room in the hospital and interviewed face‐to‐face by a trained graduate student using the constructed questionnaire. Participants were instructed to recall information for the past year. Other information, such as the treatment method, was provided by doctors/nurses, and medical bills were extracted from the healthcare information system. A total of 256 eligible participants were invited to participate in the study. Eleven FCs declined to participate due to the “lack of interest” (n = 8) or “have no time” (n = 3). Of the remaining 245 FCs who completed the questionnaire, three dyads were excluded as they had “missing data” (n = 3). Subsequently, the final sample was a total of 242 dyads and the data were used for the analysis.

### 2.4 Measurement of CHE

As recommended by the World Health Organization (WHO),^30^ CHE was defined as OOP payments for pediatric leukemia care that were greater than or equal to a threshold level of 40%^25^, ^29^, ^30^ of their households’ capacity to pay (CTP). CTP is defined as a household's non‐subsistence spending and is derived after expenses for basic needs (eg, food expenses) have been deducted from the household's expenditure^30^. According to the above variables, OOP payments for pediatric leukemia care included direct medical bills and non‐medical expenditure which were directly incurred by patients and not reimbursed by medical insurance, government subsidy, support from friends and relatives, and charity assistance. CHE was calculated based on the methods described by Wagstaff et al.^27^, ^28^

### 2.5 Statistical analysis

The demographic profile of the patients and FCs was described using the mean and standard deviation for continuous variables (eg, age, household size, and times of hospital admissions) and the frequency and percentage for categorical variables (eg, gender, residence, education, medical insurance, and disease type).

Medical expenditure, compensation, household expenditure, OOP, and CTP are presented using the mean and standard deviation to accommodate the expected skewed distributions and the proportion of these indicators was calculated where possible. The same research design from prior studies^29^, ^31^, ^32^, ^33^ was utilized in the present study, where data were further divided into four different household income groups with quartile 1 (Q1) representing the lowest income group and quartile 4 (Q4) representing the highest income group. Chi‐square analyses were performed to examine the associations between CHE and other variables including gender, age, place of residence, number of children, type of medical insurance, diagnosis, frequency of hospital admissions, therapeutic modalities, household size, government subsidy, support from relatives and friends, debt, charity assistance, non‐medical expenditure, and income level. The variables described above were statistically significant in the Chi‐square analyses (*P* < .05 in all cases). Hence, the variables were then entered into the multivariate logistic regression, with the binary indicators of “households with CHE” serving as the dependent variable. Chinese Renminbi (RMB) was converted into US dollars based on the average exchange rate (Chinese RMB 6.617 yuan = $US1.00). The entered data were then verified using the EPI Data 6.04 (EpiData Association) and analyses were performed using SPSS 23.0 (IBM Corporation).

## 3 RESULT

The complete set of descriptive statistics on the patients and their FCs demographic profiles are shown in Table 1. A total of 242 children with leukemia (age M = 8.2 years, SD = 3.9) were included in the analysis. Majority of the patients were male (61.2%), covered by health insurance (79.8%) and diagnosed as acute lymphocytic leukemia (ALL; 78.1%). The proportion of patients from urban and rural areas wAS almost equal at 48.3% and 51.7%, respectively, and the mean number of hospital admission was 3.4 times. The average debt was $6044 in a year and 8.7% of the patients underwent bone marrow transplantation. Among the 242 FCs, the mean age was 37.3 years (SD = 7.9). Majority of FCs were parents of the patients (90.9%) and had attained a junior school education or below (55.8%).

**TABLE 1:** Socio‐demographic and clinical characteristics of the participants (N = 242)

Variable	n (%)	Mean (SD)
*Characteristics of the patients*		
Gender		
Male	148 (61.2%)	
Female	94 (38.8%)	
Age		8.2 (3.9)
Place of residence		
Urban	117 (48.3%)	
Rural	125 (51.7%)	
Education		
Kindergarten or below	122 (50.4%)	
Primary school	83 (34.3%)	
Junior school or above	37 (15.3%)	
One‐child family		
Yes	161 (66.5%)	
No	81 (33.5%)	
Household size		3.6 (1.1)
Medical insurance		
MIUR	81 (33.5%)	
NCMS	68 (28.1%)	
CMI	18 (7.4%)	
MIUR/NCMS + CMI	26 (10.7%)	
Self‐payment	49 (20.2%)	
Diagnosis		
ALL	189 (78.1%)	
AML	53 (21.9%)	
Therapeutic modalities		
Bone marrow transplantation (BMT)	21 (8.7%)	
Chemotherapy	221 (91.3%)	
Frequency of hospital admission		3.4 (2.9)
Time since cancer diagnosis (day)		315.7 (425.5)
Debt		$6044 (9247)
*Characteristics of the FCs*		
Gender		
Male	85 (35.1%)	
Female	157 (64.9%)	
Age		37.3 (7.9)
Education		
Junior school or below	135 (55.8%)	
High school	55 (22.7%)	
University or above	52 (21.5%)	
Employment status		
Yes	152 (62.8%)	
No	90 (37.2%)	
Relationship to patient		
Parent	220 (90.9%)	
Other	22 (9.1%)	

Abbreviations: ALL, acute lymphocytic leukemia; AML, acute myeloid leukemia; CMI, Commercial Medical Insurance; MIUR, Medical Insurance for Urban Residents; NCMS, New Cooperative Medical Scheme.

The mean annual household expenditure and CTP were $31 256 (SD = 16 818) and $27 594 (SD = 16 252), respectively. The annual medical expenditure was $23 410 (SD = 15 603) and 94.3% of the expenses were for medical bills. The average amount of financial compensation provided to the households was $15 170. Out of this, a large proportion was from medical insurance (47.4%), followed by charity assistance (37.5%). Both medical expenditures and compensation received increased proportionally to household income, where households in the Q1 income bracket had the lowest medical expenses and compensation while households in the Q4 income bracket with the highest medical expenses and compensation. Nonetheless, the main differences lay in the source of which the households received their compensation. For households in the lower income bracket, majority of the compensation received was from compensation through medical insurance, followed by support from relatives and friends. Moreover, households in the upper income bracket received the most from charity assistance, followed by medical insurance. Urban‐rural disparities in medical expenditure, compensation, OOP payment, and CHE were also observed and a summary is presented in Table 2.

**TABLE 2:** Medical expenditure, compensation, OOP payment, and CHE by household economic status and residence

Indicators	Annual household income								Residence				Total (N = 242)	
	Q1 (n = 58)		Q2 (n = 61)		Q3 (n = 61)		Q4 (n = 62)		Urban (n = 117)		Rural (n = 125)			
	Mean (SD)	%	Mean (SD)	%	Mean (SD)	%	Mean (SD)	%	Mean (SD)	%	Mean (SD)	%	Mean (SD)	%
Medical expenditure	18 751 (11 966)	100	19 649 (12 335)	100	23 403 (15 173)	100	31 474 (18 736)	100	25 012 (14 805)	100	21 910 (16 231)	100	23 410 (15 603)	100
Medical bills	17 915 (11 389)	95.5	18 004 (11 355)	91.6	22 165 (14 455)	94.7	29 930 (17 907)	95.1	23 351 (14 202)	93.4	20 904 (15 374)	95.4	22 087 (14 839)	94.3
Non‐medical expenditure	837 (1287)	4.5	1646 (2033)	8.4	1238 (1351)	5.3	1544 (3522)	4.9	1661 (1985)	6.6	1006 (2458)	4.6	1323 (2261)	5.7
Compensation	7306 (5399)	100	7931 (5601)	100	14 828 (8050)	100	29 984 (15 664)	100	17 729 (15 971)	100	12 774 (9725)	100	15 170 (13 326)	100
Insurance reimbursement	5606 (5276)	76.7	4829 (5385)	60.9	7444 (7006)	50.2	10 727 (8358)	35.8	7800 (6634)	44.0	6611 (7299)	51.7	7186 (6997)	47.4
Government subsidy	121 (235)	1.7	115 (432)	1.4	207 (635)	1.4	23 (124)	0.1	97 (322)	0.5	133 (479)	1.0	116 (410)	0.8
Support from relatives and friends	1448 (1262)	19.8	2062 (1467)	26.0	2266 (2374)	15.3	2915 (3018)	9.7	2229 (2587)	12.6	2143 (1803)	16.8	2185 (2212)	14.4
Charity assistance	131 (416)	1.8	925 (1732)	11.7	4910 (3443)	33.1	16 320 (13 811)	54.4	7603 (12 575)	42.9	3887 (5373)	30.4	5683 (9717)	37.5
OOP payment	11 445 (8552)		11 718 (8459)		8742 (9001)		7648 (13 369)		10 262 (11 212)		9484 (9132)		9860 (10 177)	
Household income	4549 (1770)		9427 (1257)		17 280 (3099)		47 360 (29 092)		25 896 (29 206)		14 396 (10 264)		19 956 (22 315)	
Household expenditure	28 573 (15 595)	100	27 205 (13 317)	100	30 112 (14 557)	100	38 880 (20 585)	100	33 274 (15 910)	100	29 368 (17 478)	100	31 256 (16 818)	100
Food expenditure	4769 (3380)	16.7	3401 (2158)	12.5	3182 (1976)	10.6	3355 (1696)	8.6	3739 (2299)	11.2	3590 (2573)	12.2	3662 (2440)	11.7
Capacity to pay (CTP)	23 803 (13 652)	83.3	23 804 (12 508)	87.5	26 929 (14 725)	89.4	35 524 (20 245)	91.4	29 535 (15 351)	88.8	25 778 (16 912)	87.8	27 594 (16 252)	88.3
OOP share of CTP		48.1		49.2		32.5		21.5		34.7		36.8		35.7
Households with CHE		69.0		63.9		26.2		16.1		40.2		46.4		43.4

Note

Q1 represents the lowest income group and Q4 represents the highest income group.

Expenditure is standardized by gender and age.

The mean OOP payment for pediatric leukemia care was $9860, which accounted for approximately 35.7% of the household's CTP and showed a downward trend as the household income increases (Q1 = 48.1%; Q4 = 21.5%). CHE for pediatric leukemia was found to be 43.4% when measured using a 40% threshold, where the lower the household income, the higher the prevalence of CHE (Table 2).

Patients with pediatric leukemia who had medical insurance (*P* < .001), greater frequencies of hospital admissions (*P* < .001), bone marrow transplantation (*P* = .024), longer time of diagnosis (*P* < .001), government subsidy (*P* < .001), charity assistance (*P* < .001), and from a higher income group (*P* < .001) tended to have lower incidence of CHE than the others (Table 3). The logistic regression model further confirmed that medical insurance, frequency of hospital admissions, charity assistance, and income level were significant predictors of CHE (Table 4).

**TABLE 3:** Relationship between patients’ characteristics, and the prevalence of catastrophic expenditures (N = 242)

Variables	Total n	With CHE		Without CHE		*P*‐value
		n	%	n	%	
Gender						
Male	148	54	51.4	94	68.6	.007
Female	94	51	48.6	43	31.4	
Age						
1‐6	86	41	39.0	45	32.8	.329
7‐12	110	42	40.0	68	49.6	
13‐18	46	22	21.0	24	17.5	
Place of residence						
Urban	117	47	44.8	70	51.1	.329
Rural	125	58	55.2	67	48.9	
One‐child family						
Yes	161	77	73.3	84	61.3	.050
No	81	28	26.7	53	38.7	
Household size						
1‐3	139	64	61.0	75	54.7	.333
4‐7	103	41	39.0	62	45.3	
Medical insurance						
MIUR	81	36	34.3	45	32.8	.000
NCMS	68	37	35.2	31	22.6	
CMI	18	4	3.8	14	10.2	
MIUR/NCMS + CMI	26	1	1.0	25	18.2	
Self‐payment	49	27	25.7	22	16.1	
Disease type						
ALL	189	84	80.0	105	76.6	.531
AML	53	21	20.0	32	23.4	
Therapeutic modalities						
Bone marrow transplantation (BMT)	21	14	13.3	7	5.1	.024
Chemotherapy	221	91	86.7	130	94.9	
Frequency of hospital admissions						
1‐2	134	39	37.1	95	69.3	.000
3‐4	35	14	13.3	21	15.3	
≥5	73	52	49.5	21	15.3	
Time since cancer diagnosis (year)						
0‐1	162	47	44.8	115	83.9	.000
≥1	80	58	55.2	22	16.1	
Debt						
Yes	101	44	41.9	57	41.6	.963
No	141	61	58.1	80	58.4	
Government subsidy						
Yes	39	27	25.7	78	74.3	.000
No	203	78	74.3	125	91.2	
Support from relatives and friends						
Yes	237	102	97.1	135	98.5	.655
No	5	3	2.9	2	1.5	
Charity assistance						
Yes	132	29	27.6	103	75.2	.000
No	110	76	72.4	34	24.8	
Non‐medical expenditure						
0‐360	121	46	43.8	75	54.7	.092
≥360	121	59	56.2	62	45.3	
Household income						
Q1	58	40	38.1	18	13.1	.000
Q2	61	39	37.1	22	16.1	
Q3	61	16	15.2	45	32.8	
Q4	62	10	9.5	52	38.0	

Abbreviations: CMI, Commercial Medical Insurance; MIUR, Medical Insurance for Urban Residents; NCMS, New Cooperative Medical Scheme.

**TABLE 4:** Multivariate logistic regression model of determinants of CHE

Variable	B	SE	Wald	*P*‐value	OR	95% CI	
						Lower	Upper
Gender							
Male vs Female	−0.525	0.383	1.881	.170	0.592	0.279	1.253
One‐child family							
Yes vs No	0.485	0.398	1.488	.223	1.625	0.745	3.544
Health insurance							
MIUR vs Self‐payment	−0.805	0.526	2.341	.126	0.447	0.159	1.254
NCMS vs Self‐payment	0.485	0.527	0.849	.357	1.624	0.579	4.559
CMI vs Self‐payment	−0.811	0.812	0.999	.318	0.444	0.091	2.180
MIUR/NCMS + CMI VS Self‐payment	−2.672	1.136	5.534	.019	0.069	0.007	0.640
Therapeutic modalities							
BMT vs chemotherapy	0.944	0.780	1.467	.226	2.571	0.558	11.852
Frequency of hospital admission							
1‐2 vs ≥5	−1.502	0.552	7.407	.006	0.223	0.075	0.657
3‐4 vs ≥5	−0.474	0.627	0.571	.450	0.623	0.182	2.127
Time since cancer diagnosis							
0‐1 vs ≥1	−0.736	0.538	1.875	.171	0.479	0.167	1.374
Government subsidy							
Yes vs No	0.122	0.580	0.044	.833	1.130	0.363	3.518
Charity assistance							
Yes vs No	−1.210	0.491	6.078	.014	0.298	0.114	0.780
Household income							
Q1 vs Q4	1.484	0.685	4.696	.030	4.412	1.152	16.891
Q2 vs Q4	1.911	0.620	9.511	.002	6.763	2.007	22.787
Q3 vs Q4	0.567	0.557	1.039	.308	1.764	0.592	5.251

## 4 DISCUSSION

In the present study, the incidence of CHE for pediatric leukemia was 43.4%. This proportion was similar to a recent study conducted, where the authors found the incidence of CHE for six types of cancers including esophageal, colon, gastric, liver, lung, and breast cancer to be 42.78%.^20^ The prevalence of CHE was also similarly reported in prior studies conducted in Malaysia (47.8%),^24^ South Korea (39.8%),^15^ and eight Southeast Asian countries (48%).^19^ However, this proportion was much lower than in another study conducted in China. A possible reason for this is that in their study, almost 95% of the families were living with end‐of‐life cancer, thus explaining the higher incidence of CHE.^21^ It should be noted, however, that there are several methodological differences between the present study and above‐mentioned studies. With the exception of the study that was conducted in Malaysia,^24^ all of the previous studies included medical bills in their calculation of OOP payments and hence, may have influenced the incidence of CHE. Furthermore, these studies only examined medical insurance as the source of compensation rather than omitting other forms of financial aid such as charity assistance, and this may have further overestimated the incidence of CHE. In addition, the threshold used to define CHE varied across the studies, ranging from 10%,^15^ 30%^19^ and 40%.^20^, ^21^, ^24^ In light of the possible shortcomings in previous studies, non‐medical expenses and other sources of compensation, such as charity assistance, were included in the calculation of CHE in the current study. Nonetheless, results from both the current study and previous studies indicate that there is a potential risk for leukemia to result in significant financial and economic burden. The high incidence of CHE in households living with pediatric leukemia could also be due to the finding that the majority of the patients were the only child of the family (66.5%). As treatment and prognosis of leukemia have been improving, the family would, therefore, be more likely to ensure continued treatment and finance for the patient's medical expenses. This is supported by the response from the interview and the finding that the average debt for households living with pediatric leukemia is $6044, hence the higher prevalence of CHE in pediatric leukemia.

It is clear that the past literature that households with strong financial capacity would serve as an effective buffer in the prevention of CHE, irrespective of a country's economic development.^15^, ^19^, ^20^, ^21^, ^24^, ^28^, ^34^, ^35^ For example, in developed countries, a study conducted in South Korea found that the prevalence of CHE among households with lower income was higher than those with higher income ^15^. Similar results were also found in developing countries such as China,^21^ Malaysia,^24^ and other Southeast Asian countries.^19^ Consistent with the above‐mentioned studies, economic status was also found to play a key role in the prevalence of CHE in the current study, with households in the lower income bracket to be more likely to experience CHE. In light of the notion that income inequality may have exacerbated the prevalence of catastrophic OOP payments for the households with lower income, it is pertinent that the disparity of income distribution be addressed.

To the authors’ knowledge, there was no previous study conducted on the association of CHE and charity assistance. However, charity assistance was the second most frequent form of health expenditure compensation in the treatment of pediatric cancer.^36^ There were several methods to which families living with leukemia in the present study were able to receive charity assistance. First, charitable organizations, various foundations, and non‐profit organizations are often dedicated to provide financial assistance for children with leukemia. Second, traditional media, such as television, radio, and paper media, would often disseminate information to raise awareness on pediatric leukemia and help to raise funds. Finally, with greater accessibility to the Internet, family members of children with leukemia can now easily raise funds online through crowdfunding. However, in the present study, findings showed that the amount of charity assistance received by a household is inversely proportionate to household income. On average, families in Q1 received $131 in charity assistance whereas families in Q4 received $16 320. This is inconsistent with previous studies which found that households with lower income are more likely to receive greater charity assistance than those with higher income.^37^ A possible reason for this discrepancy could be due to the inequitable utilization of charity assistance that may exist across household groups. These trends suggest the need to widen the lens on inequitable utilization of charity assistance and remove any potential barriers that may affect households from accessing these funds.

Health insurance is commonly perceived as an important means to avoid CHE.^29^, ^35^ However, households that were covered only by a single medical insurer, such as MIUR, NCMS or CMI, were also found to be more likely to experience CHE. This finding is consistent with the results of a recent study on CHE in the treatment of cancer.^21^ One explanation is the low payout and compensation rate for MIUR and NCMS,^20^, ^21^, ^31^ and CMI is a relatively new medical coverage.^38^ However, for households that have both the basic medical insurance (either MIUR or NCMS) and CMI, they are less likely to experience CHE. This could be attributed to these households obtaining a greater amount of compensation for having both medical coverages. In addition, compared to households with higher income, households with lower income are more likely to rely on insurance to provide aid for their health expenditure (76.1% in Q1 and 35.8% in Q4). This could be due to disparity in economic status, or households with lower income seeking alternate sources of financial aid and thus, the lower proportion of compensation from medical insurance relative to their higher income counterparts. Addressing the challenges faced by families experiencing CHE requires the development of a multi‐tiered medical insurance system. This may include medical insurance as the basic level of coverage, while other forms of insurance, such as commercial health insurance and catastrophic health insurance, could be added to expand the scope and depth of compensation so as to reduce the OOP medical payments.^21^, ^39^

Similar to an earlier study,^40^ the current study revealed that patients with a history of frequent hospital admissions are more likely to report greater economic burden from OOP payments and CHE. Leukemia requires repeated hospitalizations for treatment.^41^ Hence, in terms of health services and treatment habits,^42^, ^43^ FCs preferred more specialized medical institutions to obtain better treatment for their loved ones with pediatric leukemia. Repeated hospitalizations require families, especially those living in rural areas, to bear more non‐medical costs such as transportation and accommodation, which may not be covered by medical insurance. With greater frequency of hospital admissions, this would translate to a higher financial cost and thus, greater economic burden for households living with pediatric leukemia.

### 4.1 Limitation

The sample comprised of 242 child‐caregiver dyads seeking treatment for pediatric leukemia in the region, which may limit its generalizability to patients with other pediatric oncology. Therefore, it is recommended for future studies to recruit a larger sample from multiple regions. As some of the self‐reported measures, such as annual household income and expenditure, required participants to recall information in the past year, there may have been recall biases which could have affected the results. In order to minimize recall bias, data were, therefore, collected not only from the FCs and doctors/nurses, but also through the health information system wherever possible. In addition, precautionary measures were taken in the calculation of OOP payments using comprehensive cost obtained from both medical bills and non‐medical expenses after accounting for financial assistance through compensation such as medical insurance, subsidies, charity donations, and support from relatives and friends. In the present sample, patients were seeking bone marrow transplantation and chemotherapy, and CHE for other forms of therapeutic modalities was not examined. Novel types of immunotherapy, such as Chimeric antigen receptor‐engineered T cells (CAR‐T cells), should be explored in future studies.

## 5 CONCLUSION

The results revealed that pediatric leukemia can have a significant catastrophic effect on families, especially for those with a low economic status. The occurrence of CHE in households living with pediatric leukemia could be reduced by addressing income disparity. In addition, extending coverage and improving compensation from medical insurance could also alleviate CHE. Some other measures that can be implemented to minimize CHE are to address the barriers of charity assistance for vulnerable groups.

## CONFLICT OF INTEREST

All authors declare no potential competing interests.

## AUTHOR CONTRIBUTIONS

Conceptualization, Xueyun Zeng and Hongjuan Yu; Data curation, Mingjie Sui, Xueyun Zeng, Rui Liu, Bo Liu, and Wenrui Ma; Funding acquisition, Weidong Huang and Hongjuan Yu; Methodology, Weidong Huang, Mingjie Sui, and Sihai Tao; Writing – original draft, Weidong Huang, Mingjie Sui, WanJie Tan, and Hongjuan Yu; Writing – review & editing, Weidong Huang, WanJie Tan, and Hongjuan Yu. All authors read and approved the final manuscript.

## Data Availability

The data of the current study are available from the corresponding authors upon reasonable request.
