# Supplementary material for: Catastrophic health expenditures of households living with pediatric leukemia in China
Source: Cancer Med. 2020 Jul 22;9(18):6802–12. doi: 10.1002/cam4.3317 (PMC7520357; doi:10.1002/cam4.3317)
Supplement: Supplementary file 1 [file CAM4-9-6802-s001.docx]

**Financial burden of households with pediatric leukemia**

**1. Demographic profile**

Conditions of the patient

1). Gender: ( ) ①Male ②Female

2). Age: ( ) years old

3). residence: ( ) ①Urban ②Rural

4). Education level: ( ) ①Kindergarten or below ②Primary school ③Junior school or above

5). Ethnicity: ( )

6). Religious belief: ( )

7). Medical insurance of patient: ( )

caregiver

8). Gender: ( ) ①Male ②Female

9). Age: ( ) years old

10). Relationship with the patient who you care for: ( ) ①Parents ②Other

11). Ethnicity: ( )

12). Religious belief: ( )

13). Education level: ( ) ①Junior school or below ②High school ③University or above

14). Marital status: ( ) ①Married ②Unmarried Married ③Divorced ④Widowed

15). Employment: ( ) ①Employed ②Retired ③Unemployed

16). How long have you been caring for your patients: ( )months ( ) hours per day

**2. Household information**

1). Household size: ( )

2). Household income in previous year ( )

3). Household debt in previous year ( )

4). Household expenditure in previous year ( )

| Household expenditure (over the year) |  |
| --- | --- |
| Food |  |
| Medical service |  |
| Transportation and communication |  |
| Accommodation |  |
| Education |  |
| Amusement |  |
| Daily necessities |  |
| Other |  |

**3. Financial cost of cancer treatment**

Medical expenditure over the year ( )

| Medical expenditure (over the year) |  |
| --- | --- |
| Medical bills |  |
| Non-medical expenditure |  |
| Transportation |  |
| Accommodation |  |
| Nutrition food |  |

**4. Compensation**

| Compensation (over the year) |  |
| --- | --- |
| Insurance |  |
| Government subsidy |  |
| Support from relatives and friends |  |
| Charity assistance |  |

**5. Clinical information.**

1). Name of disease: ( )

2). Therapeutic modality: ( )

3). Date of diagnosis: ( )year ( )month ( )day

4). Frequency of hospital admission (over the year): ( )
